# *Nilonema gymnarchi* (Nematoda: Philometridae) and trace metals in *Gymnarchus niloticus* of Epe lagoon in Lagos State, Nigeria

**DOI:** 10.1016/j.heliyon.2020.e04959

**Published:** 2020-09-28

**Authors:** Patrick Omoregie Isibor, Bamidele Akinsanya, Temitope Sogbamu, Fatsuma Olaleru, Akeredolu Excellence, Benjamin Komolafe, Saliu Joseph Kayode

**Affiliations:** a Department of Biological Sciences, Covenant University, PMB 1023, Ota, Ogun State, Nigeria; b Department of Zoology, University of Lagos, P.O. Box 156, Akoka, Nigeria

**Keywords:** Environmental analysis, Environmental assessment, Environmental impact assessment, Environmental pollution, Environmental risk assessment, Environmental toxicology, Toxicology, Zoology, Bioaccumulation, Trace metals, Health risk, Toxicity, Histopathology, Enteric parasite

## Abstract

The presence of trace metals in the sediment, water, and biota of the Epe lagoon has been recently linked to oil exploration and municipal perturbations around the lagoon. The study was aimed at assessing the concentrations and associated health risks of Fe, Zn, Cu, Ni, Pb, Cd, Cr, Mn, Co and V in the water, sediment, and *Gymnarchus niloticus* of Epe lagoon and to evaluate the role of the enteric parasite *Nilonema gymnarchi* in bioaccumulation of the metals in the fish. The temperature, pH, redox potential, conductivity, turbidity, dissolved oxygen (DO), total dissolved solids (TDS), and salinity were determined in-situ using a handheld multi-parameter probe (Horiba Water Checker Model U-10). The concentrations of Fe, Zn, Cu, Ni, Pb, Cd, Cr, Mn, Co, and V were determined in the surface water, bottom sediment, *Gymnarchus niloticus,* and its enteric parasites, *Nilonema gymnarchi* in Epe lagoon using the Flame Atomic Absorption Spectrometer (Philips model PU 9100). The bioaccumulation factors and target hazard quotients of the trace metals in the infected and uninfected fish were estimated and compared. The intestinal tissue sections of the infected and uninfected fish were examined using a binocular dissecting microscope (American Optical Corporation, Model 570) using hematoxylin and eosin (H&E) stain. Biochemical markers such as reduced glutathione (GSH), superoxide dismutase (SOD), catalase (CAT), and lipid peroxidation (MDA) were determined in the liver of the infected and uninfected fish. The SOD level was higher in the uninfected fish than the infected ones supports the indications deduced from the bioaccumulation analysis. Strong positive correlations between SOD and most of the metals- Fe (0.916), Zn (0.919), Cu (0.896), and Ni (0.917) suggests that the metals may have inflicted more toxicity in the uninfected. The histopathological comparisons made between the uninfected and infected fish showed consistency with the outcomes of other comparisons made in this study. These evidence were marked by tissue alterations in the infected fish ranging from no observed changes to mild alterations, while the uninfected exhibited more severe tissue injuries such as hemorrhagic lesions, severe vascular congestion, edema, the increased connective tissue of the submucosa, and vascular congestion. The condition factors of the infected (0.252) and uninfected (0.268) fish indicated slenderness and unfitness possibly due to environmental stressors such as trace metals. The parasitized fish showing better-coping potentials than the uninfected, coupled with the significant bioaccumulation interferences exhibited by the parasite *Nilonema gymnarchi* is an indication that the parasites may be a good metal sequestration agent for the fish and can be used to forestall the significant health hazard quotient posed by the current level of iron and the synergy of all metals analyzed in the lagoon.

## 1 Introduction

Predominant oil exploration activities around Epe lagoon are primarily responsible for the attendant oil spill incidences at the lagoon. Previous reports have linked the use of petroleum products, combustion of fossil fuels, and tons of municipal wastes to pollution of the lagoon (Doherty and Otitoloju, 2016; Akinsanya et al.*,* 2019; Isibor et al., 2020). This pollution has been characterized by poor water quality and its impact on the sentinel species in the aquatic ecosystem (Akinsanya et al.*,* 2019). The presence of trace metals in the sediment, water, and biota of the lagoon has also been previously linked to the operations of oil industries within the catchment area (Smith and Guentzel, 2010; Enuneku and Ilegomah, 2015; Akinsanya et al., 2019).

The bioaccumulation of trace metals in the different fish tissues with toxic effects marked by a change in the physiological activities and biochemical composition of the fish tissues has been widely reported (Gad and Yacoub, 2009; Cao et al., 2010; Malik et al., 2010; Jovanovic et al., 2011; Ebrahim and Taherianfard, 2011; George et al., 2013; Olawusi-Peters et al., 2014; Saliu and Akinsanya, 2014; Ukwa et al.*,* 2015). Bawuro et al. (2018) investigated the bioaccumulation of metals in selected tissues of *Heterotis niloticus* and *Tilapia zilli* in Lake Geriyo, Adamawa State, Nigeria. Their result showed that metal accumulation varied depending on species-specificity, feeding behavior, fish size, and age.

The combination of histological studies and analysis of the biochemical profile in fish has proven to be efficient tools in the evaluation of metal toxicity (Kaoud and El-Dahshan, 2010; Mahino and Nazura, 2013; Akinsanya et al.*,* 2019). Histopathological alterations can be used as indicators for the effects of various contaminants, including trace metals on aquatic biota especially fish, and are a reflection of the overall health of the entire population in the ecosystem (Mohamed, 2009; El-Bakary et al., 2011).

An extensive amount of literature exists on the use of histopathology as a tool for monitoring trace metals in aquatic ecosystems (Aly et al.*,* 2003; Van Dyk, 2003; Mela et al.*,* 2007; Atif et al.*,* 2009; Otitoloju et al., 2009; Kaoud and El-Dahshan, 2010; Mahino and Nazura, 2013; Nsofor et al., 2014; Alimba et al.*,* 2015; Abalaka, 2015; Amuno et al., 2016).

The biochemical biomarkers, on the other hand, are considered to be early indicators of toxicity in fish and have shown promising results in both the field and the laboratory studies (Geoffroy et al., 2004; Ubani-Rex and Saliu et al., 2017), thus gaining substantial scientific credence (McFarland et al., 1999; Wedderburn et al., 2000; Lionetto et al., 2003; Pandey et al., 2003; Farombi et al., 2007; El-Gazzar et al., 2014; Ubani-Rex and Saliu et al., 2017).

*Gymnarchus niloticus* (Curie, 1892) is a common fish species in Nigeria and several West African countries. It is the only member of the family *Gymnarchidae* in the order of Osteoglossiformes. The fish species live in a demersal, potamodromous, freshwater environment with a pH range of 6.5–8.0 (Riede, 2004). The species occur widely in the Nile, Volta, Chad, Senegal, Gambia basins and Lake Rudolf. *Gymnarchus niloticus* is one of the dominant species of fish in the lagoon that is yet to be fully studied. The fish is not only economically important, it is also of great socio-cultural importance in Nigeria (Ayoola and Abotti, 2010; Oladosu et al., 2011) which endears it as one of the most highly valued freshwater fishes in Nigeria. Despite its aquaculture potentials including rapid growth, high premium, tasteful, seasonal availability of wild growers (Kigbu et al., 2014), the supply of *Gymnarchus niloticus* relies greatly on the wild collection which is exposed to trace metal contamination and might be unfit for consumption (Andrew et al., 1994).

Studies have shown that exposure of fish to xenobiotics may compromise their immune system, thereby making them more susceptible to parasitic infections (Akinsanya et al. 2020). Conversely, other studies have shown that parasites may also relieve toxicant burdens in their fish hosts (Akinsanya et al.,2019). A trade-off point exists between parasite morbidity and depuration capacity in the host (Akinsanya et al.*,* 2020).

The study was aimed at assessing the concentrations and associated health risks of Fe, Zn, Cu, Ni, Pb, Cd, Cr, Mn, Co and V in the water, sediment, and *G. niloticus* of Epe lagoon. It also seeks to evaluate the role of the enteric parasite *Nilonema gymnarchi* in bioaccumulation of the metals in the liver and intestine of the fish.

## 2 Materials and methods

### 2.1 Description of the study area

**Figure 1:**
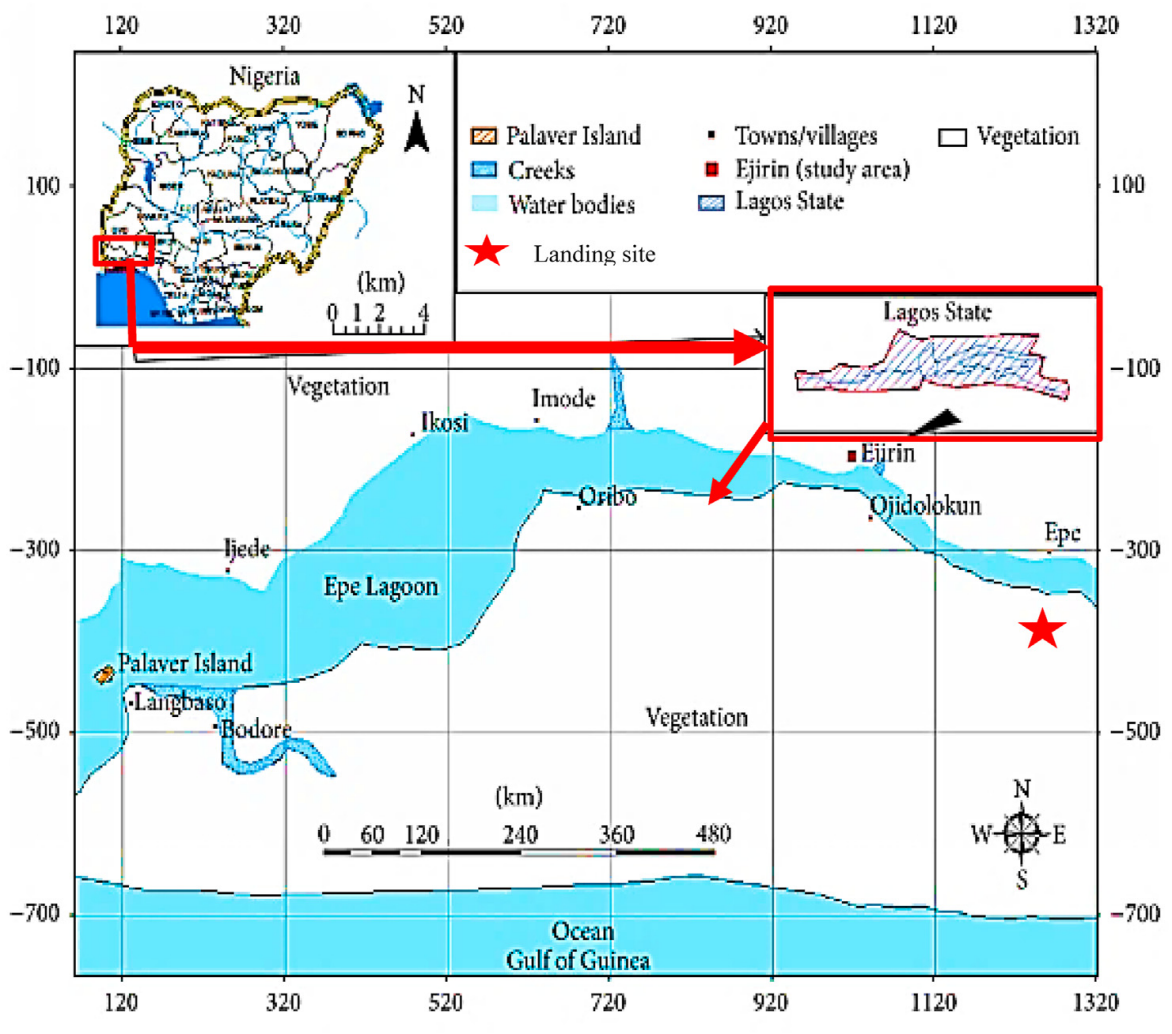
Map of the study area. Map designed using QGIS software version 3.10.1 ′A Coruña' (QGIS Development Team, 2019). URL: https://qgis.org/en/site/forusers/download.html#.

Epe lagoon is located in Lagos State, South-Western Nigeria (Figure 1). The Lagoon is one of the major lagoons in Lagos State, Nigeria (Kumolu-Johnson et al., 2010). The other lagoons found in Lagos State include Ologe Lagoon and Lagos Lagoon. The lagoon is situated to the east, Lagos Lagoon in the central and Ologe Lagoon is situated to the west. The lagoon lies between latitudes 6°29 'N and 6°38 'N; and longitudes 3°30 'E and 4°05 'E. It has a surface area of about 247 km^2^ with a maximum depth of 6.4 m; a greater part of the lagoon is shallow and less than 3.0 m deep (Akinsanya and Adekogbe, 2017). It is fed by River Oni discharging to the North-Eastern sections and Rivers Oshun and Saga discharging into the North-Western sections of the Lagoon.

Epe Lagoon is part of an intricate system of waterways made up of lagoons and creeks that are found along the coast of South-Western Nigeria from the Dahomey border to the Niger Delta stretching over a distance of about 200 km (Agboola and Anetekhai, 2008). The lagoon is very important because of the nursery and breeding ground it provides for a large variety of fish. Due to increased human activities and speedy industrialization, there has been a high discharge of industrial effluent containing trace metals into the Lagoon (Akinsanya et al., 2019, Akinsanya et al., 2020).

The vegetation around the lagoon is characterized by shrubs and Raphia Palms (*Raphilia sudanica)* and Oil Palms (*Elaies guineensis*). Floating grass occurs on the periphery of the lagoon while coconut palms *(Cocus nicifera)* are widespread in surrounding villages. The lagoon experiences both dry and rainy seasons typical of the Southern part of Nigeria.

The rich fauna of the lagoon according to Kusemiju (1981) include; *Heterotis niloticus* (Ehenberg, 1929)*, Gymnarchus niloticus* (Cuvier, 1829)*, Clarias gariepinus* (Burchell, 1822)*, Malapterurus electricus* (Forskal, 1775)*, Synodontis clarias* (Linnaeus, 1762)*, Chrysichthys nigrodigitatus* (Lacepede, 1802)*, Parachanna, obscura* (Gunther, 1861), *Mormyrus rume* (Boulenger, 1898)*, Calabaricus calamoichthys* (Smith, 1865)*, Tilapia zillii* (Gervais, 1848)*, Tilapia galilaea* (Artedi, 1757)*, Hemichromis fasciatus* (Peters, 1865) *and Sarotherodon melanotheron* (Ruppel, 1852).

### 2.2 Sample collection

All samples were collected for a period of 9 months (February–September 2018) on the 15^th^ day of every month. The temperature, pH, redox potential, conductivity, turbidity, dissolved oxygen (DO), total dissolved solids (TDS), and salinity were determined in-situ using a handheld multi-parameter probe (Horiba Water Checker Model U-10) in triplicates on a monthly basis.

Surface water and bottom sediment samples were collected in triplicates, on a monthly basis. Surface water samples were collected with sterile sampling bottles, stored in an ice chest and transported immediately to the laboratory for the determination of the trace metals. Sediment samples were collected using a sterile Van Veen grab sampler of dimension 15 × 15 × 12 cm (8.5 kg). After sampling, the samples were packed in sterile polyethylene bags, preserved in a sterile ice chest, and transported immediately to the laboratory for analysis.

A total of 80 specimens of *Gymnarchus niloticus* from Epe lagoon were procured lifeless but fresh from local fishermen at the landing site of the lagoon within the period of sampling. The fishermen employed local fishing tools such as hooks and lines, nets, and traps in the fish capture. The fish samples were dissected using clean dissecting kits sterilized with saline water to expose and transfer the intestinal and liver tissues into a petri dish. The intestines were removed from the Petri dishes containing the saline water and the portion for histopathological examination was placed differently in a sample bottle containing Bouin's fluid while the one for metal analysis was in another sample bottle preserved in 70% alcohol. The liver was also equally placed in another sample bottle preserved in 70% alcohol.

### 2.3 Laboratory analysis

#### 2.3.1 Identification and morphometric assessment of fish samples

The fish specimens were identified to the genus levels using taxonomic keys described by Olaosebikan and Raji (1998), and Idodo-Umeh (2003).

The weight and standard length of the fishes were measured in triplicates using a standard top-loading Denward Balance (Model - TX3202L-V) and meter rule respectively. The sex of the fish was determined based on the presence of testes and ovaries in the males and females respectively.

The length-weight (log-transformed) relationships were determined by linear regression analysis and scatter diagrams of length and weight were plotted. The length-weight relationship of the experimented fish was defined as described by Le Cren (1951).

W = aL^b^

Where, W = weight of fish (g), L = standard length of fish (cm), ‘a’ is the regression intercept and ‘b’ is the regression slope.

The values of ‘a’ and ‘b’ were determined from the transformed logarithm values of length and weight using the equation Log W = Log a + b Log L with the aid of Microsoft Excel software (2010).

The Fulton condition factor (k) which is an indication of the degree of robustness or the state of wellbeing of an individual organism was calculated according to Htun-Han (1978).

$$\text{K}=\frac{\text{W}\times 100}{\text{L}3}$$

W = weight of fish (g), L = Length of fish (cm).

#### 2.3.2 Examination and identification of parasites in fish

Fish specimens were dissected and the samples of intestine were collected with the aid of sterile blades and forceps (Sures, 2004). The intestines were kept in physiological saline solution, longitudinally excised and the gastrointestinal helminth parasites were collected, sorted according to groups (cestodes and nematodes). The parasites were preserved and fixed in 70% alcohol, sealed thoroughly in ethylene diamine tetra-acetic acid bottles appropriately, and identified using identification manuals such as Colombo et al. (2005), Xing et al. (2005), and Sures (2007) and Akinsanya et al. (2008).

The fish specimens were grouped into infected and uninfected based on the presence or absence of the intestinal parasites respectively.

#### 2.3.3 Trace metal analysis

##### 2.3.3.1 Analysis of metals in water

25 mL of the preserved water sample was measured and poured into PTFE (Polytetrafluorethylene) beaker and transferred into a fume cupboard and 10 mL of nitric acid was added to each sample in the beakers. These mixtures were then heated on a hot plate to the lowest volume possible (10 mL). They were allowed to cool and then filtered and made up with distilled water into 50 mL volumetric flask. The digested samples were then taken to Flame Atomic Absorption Spectrometer for analysis to measure the concentration of 10 metals; Fe, Zn, Cu, Ni, Pb, Cd, Cr, Mn, Co and V in the water sample. These readings were done triplicate for assurance of precision.

##### 2.3.3.2 Analysis of metals in sediment

The sediment sample was air-dried and sieved through 25 μm mesh. Then 1 g was weighed with the aid of a weighing balance (Model - TX3202L-V), homogenized, and was transferred into a PTFE conical flask. 25 mL of ratio 3:1 Hydrochloric and Nitric acid (aqua regia) were added to each of the samples in a fume cupboard for digestion. This was then heated on a hot plate until the volume reduced to about 5 mL. They were filtered and made up with distilled water to 50 mL volumetric flask for the trace metal concentration analysis of Fe, Zn, Cu, Ni, Pb, Cd, Cr, Mn, Co, and V, using the Flame Atomic Absorption Spectrometer (Philips model PU 9100).

##### 2.3.3.3 Analysis of metals in biota: implication for bioaccumulation and health risk

Frozen liver and intestine of the fish were thawed and two (2) grams wet-weight samples of liver and intestine (from both infected and uninfected fish) were weighed, and the enteric parasites were separately pulled to obtain the same weight. These samples were separately placed in a beaker and digested with 25 mL of ratio 1:1 hydrogen peroxide and Nitric acid. The mixture was heated to about 5 mL and allowed to cool afterward. It was then filtered and made up with distilled water to the 50 mL. Flame Atomic Absorption Spectrometer (Philips model PU 9100) was then used in analyzing the concentrations of Fe, Zn, Cu, Ni, Pb, Cd, Cr, Mn, Co and V with detection limits of 0.5 μg g^−1^, 0.01 μg g^−1^, 0.01 μg g^−1^, 0.03 μg g^−1^, 0.1 μg g^−1^, 0.05 μg g^−1^, 0.1 μg g^−1^, 0.05 μg g^−1^, 0.01 μg g^−1^, and 0.5 μg g^−1^ respectively. All procedures were guided by the guidelines of Whiteside (1981).

The bioaccumulation factor was evaluated for the 10 trace metals investigated in the parasites, intestine, and liver tissues of the infected and uninfected fish. BAF is computed as the ratio of the concentration of a pollutant accumulated in the tissue of an organism concerning the concentration of that pollutant in the water body (Authman and Abbas, 2007).

The bioaccumulation factor (BAF) for the metals in both infected and uninfected fish was calculated thus;

$$\text{BAF}=\frac{\text{Concentration of metal in fish }\left(\text{mg/kg}\right)}{\text{Concentration of metal in water }\left(\text{mg/L}\right)}$$

The bioaccumulation factor (BAF_p/i_) for the metals partition from the fish intestine to parasites was calculated thus;

$${\text{BAF}}_{\text{p/i}}=\frac{\text{Concentration of metal in parasite }\left(\text{mg/kg}\right)}{\text{Concentration of metal in intestine }\left(\text{mg/kg}\right)}$$

Biota-sediment accumulation factor (BSAF) of the metals was calculated thus;

$$\text{BSAF}=\frac{\text{Concentration of metal in fish }\left(\text{mg/kg}\right)}{\text{Concentration of metal in sediment }\left(\text{mg/kg}\right)}$$

The target hazard quotient (THQ) was adopted for health risk analysis as described by the United States Environmental Protection Agency (USEPA, 2011).

The THQs were calculated separately for the infected and uninfected fish groups thus;

$$THQ=\frac{Efr\times ED\times FIR\times C\times 10^{-3}}{RfDo\times BWa\times ATn}$$

Efr = exposure frequency (365 days), ED = exposure duration (52 years-adopted average lifespan), FIR = fish ingestion rate (5 g/day), C = concentration of metal in fish, ATn = average exposure time for non-carcinogen (365 days/year ×exposure years- 52 years), BWa = average adult body weight (70 kg was adopted), and RfDo = oral reference dose (mg/kg/day).

The reference oral doses for zinc = 0.0006, cadmium = 0.001, vanadium = 0.004, iron = 0.001, copper = 0.001, nickel = 0.001, cobalt = 0.0006, lead = 0.004, chromium = 1.5, and manganese = 0.001 were adopted from USEPA, 2010, USEPA, 2011.

Due to the likelihood of synergistic/antagonistic interactions among the metals, the total target hazard quotient (∑THQ) was considered as the sum of the target hazard quotients of every metal analyzed (Isibor et al. 2020), which was calculated thus;

∑THQ = HQ_1_ + HQ_2_ + HQ_3_. (IARC, 1983; NRC, 1983)

#### 2.3.4 Histopathological examination

The Bouin's fluid in the preserved specimens was decanted after 6 h while 10% of phosphate-buffered formalin was added to preserve the tissue. Random selection was made from the preserved tissues for analysis. The selected tissue was routinely dehydrated in an ascending series of alcohol at 30 min interval; it was then embedded in molten paraffin wax and allowed to solidify. The blocked tissues were sectioned at 4–5 microns processed and stained with hematoxylin and eosin (H&E) stains. The stained tissues were washed off in tap water. The tissues were then mounted using DPX mountant dried an examined under the binocular dissecting microscope (American Optical Corporation, Model 570) at the pathology laboratory of the department of veterinary pathology, university of Ibadan, Nigeria where the samples were taken for analysis and recording.

#### 2.3.5 Biochemical analysis

Some samples of the fish liver (15g wet weight) were weighed into a crucible then macerate and homogenized, then 10g of the homogenized tissue was placed in a 50 mL centrifuge tube, 15mL of 6N KOH was added and the tubes were incubated for 18h in a 35 °C water bath. The mixture was shaken agitated for 30 s half-hourly for 4 h and then allowed to cool.

##### 2.3.5.1 Determination of superoxide dismutase (SOD) activity

Total SOD activity in the liver tissue homogenates was determined following the procedure of Marklund and Marklund (1974) with some modifications. The method is based on the ability of SOD to inhibit the autoxidation of pyrogallol. In 970 μL of buffer (100 mMTris-HCl, 1 mM EDTA, pH 8.2), 10 μL of homogenates and 20 μL pyrogallol were mixed. The assay was performed in thermostat cuvettes at 25 °C and changes of absorption were recorded by a spectrophotometer (Spectronic 20D) at 480 nm. One unit of SOD activity was defined as the amount of enzyme that can inhibit the auto-oxidation of 50% of the total pyrogallol in the reaction.

##### 2.3.5.2 Determination of catalase (CAT) activity

Catalase (CAT) was assayed calorimetrically at 620nm and expressed as moles of hydrogen peroxide (H_2_O_2_) consumed/min/mg protein as described by Quinlan et al. (1994). The reaction mixture (1.5 mL) contained 1.0 mL of 0.01M pH 7.0 phosphate buffer, 0.1 mL of Plasma and 0.4 mL of 2M H2O2. The reaction was stopped by the addition of 2.0 mL of dichromate-acetic acid reagent (5% potassium dichromate and glacial acetic acid were mixed in 1:3 ratio).

##### 2.3.5.3 Determination of reduced glutathione (GSH) activity

Reduced glutathione (GSH) was determined by the method of Ellman (1959). To the homogenate was added 10% TCA, centrifuged. 1.0 ml of supernatant was treated with 0.5 ml of Ellmans reagent (19.8 mg of 5, 5′-dithiobisnitro benzoic acid (DTNB) in 100 ml of 0.1% sodium nitrate) and 3.0 ml of phosphate buffer (0.2M, pH 8.0). The absorbance was read at 412 nm.

##### 2.3.5.4 Determination of lipid peroxidation

Lipid peroxidation as evidenced by the formation of malondialdehyde (MDA) was measured according to the method of Niehaus and Samuelsson (1968) and Jiang et al. (1992). In brief, 0.1 mL of tissue homogenate (Tris-HCl buffer, pH 7.5)/serum was treated with 2 mL of (1:1:1 ratio) TBA-TCA-HCI reagent (thiobarbituric acid 0.37%, 0.25N HCI and 15% TCA) and placed in a water bath for 15min, cooled and centrifuged at room temperature for 10 min at 3,000 rpm. The absorbance of clear supernatant was measured against reference blank at 535 nm.

#### 2.3.6 Quality control and quality assurance

Readings were taken in triplicates to minimize errors. Reagents used were annular grades and all apparatuses used were sterilized under an autoclave (Systec HX-65) at 120 °C for 2 h. The limits of quantification (LOQ) for the metals were estimated as the fraction of the calculated and the reference mass fractions (calculated value: reference value) in the calibration curves. The LOQs for Fe, Zn, Cu, Ni, Pb, Cd, Cr, Mn, Co and V in fish tissue were 0.013, 0.012, 0.016, 0.012, 0.014, 0.017, 0.013, 0.014, 0.013, and 0.014 mg.kg^−1^respectively, which were validated using certified reference materials (CRMs) such as TraceCERT® and ERM-CE27. Adequacy of trueness was evaluated using statistical tool z-scores which were used to determined standard deviations from the certified reference materials at < 6% and 95% confidence interval.

### 2.4 Statistical analysis

The descriptive statistics of the concentrations of trace metals in the water, sediment and fish tissues were presented as mean ± standard deviation, which was subjected to analysis of variance (ANOVA) to determine the significant differences using Microsoft Excel (2010) and SPSS (version 20). The actual locations of the significant differences were further determined by Tukey post-hoc test. All statistical analyses were conducted at a probability level of 0.05.

## 3 Results

### 3.1 Physiochemical parameters of Epe lagoon

The pH of the surface water samples from Epe lagoon was lower than the established range by FEPA suggesting the water was acidic (Table 1). The turbidity which ranged from 132- 235 NTU was markedly higher than the regulatory limit set at 5 NTU throughout the 9 months of study. Other physicochemical parameters were below the established limits.

**Table 1:** Physicochemical properties of surface water from Epe lagoon.

Parameters	Mean ± SD	Min	Max	FEPA (2003)
Temperature (°C)	30.12 ± 2.68	25.49	32.54	–
pH	**4.48 ± 0.12**	4.30	4.66	6.5–8.5
Redox Potential (ORPmV)	398.00 ± 38.04	340.00	454.00	–
Conductivity (μS/cm)	42.89 ± 28.58	3.00	80.00	–
Turbidity (NTU)	**173.33 ± 38.29**	132.00	235.00	5
Dissolved Oxygen, DO (mg/l)	22.91 ± 9.68	12.46	38.53	>7.5
Percentage DO (%)	277.53 ± 79.74	167.40	374.80	–
Total Dissolved Solids (g/L)	7.67 ± 6.24	1.00	16.00	2000

Emboldened figures are higher/lower than established standard limits. Sample size (N) = 27.

### 3.2 Morphometrics of *Gymnarchus niloticus*

Of the total 80 specimens of *Gymnarchus niloticus* examined, the infected fish (830.80 ± 686.6 g; 65.5 ± 16.9 cm) had a mean condition factor of 0.25 ± 0.07 (Table 2). The uninfected fish (477.64 ± 285.1 g; 55.4 ± 9.1 cm) on the other hand had a mean condition factor of 0.27. Results indicate that both condition factors were poor (<1).

**Table 2:** Morphometries and length-weight relationship of *G. niloticus*.

	*n*	Standard length (cm)			Weight (g)			Condition Factor (k)			b
		Mean ± SD	Min	Max	Mean ± SD	Min	Max	Mean ± SD	Min	Max	
Infected	69	65.5 ± 16.9	40.1	110.6	832.80 ± 26.6	211	2673	0.2524 ± 0.07	0.13	0.38	2.74
Uninfected	11	55.4 ± 9.1	45	73.6	477.64 ± 25.1	258	1295	0.2680 ± 0.05	0.16	0.36	2.45

**Figure 2:**
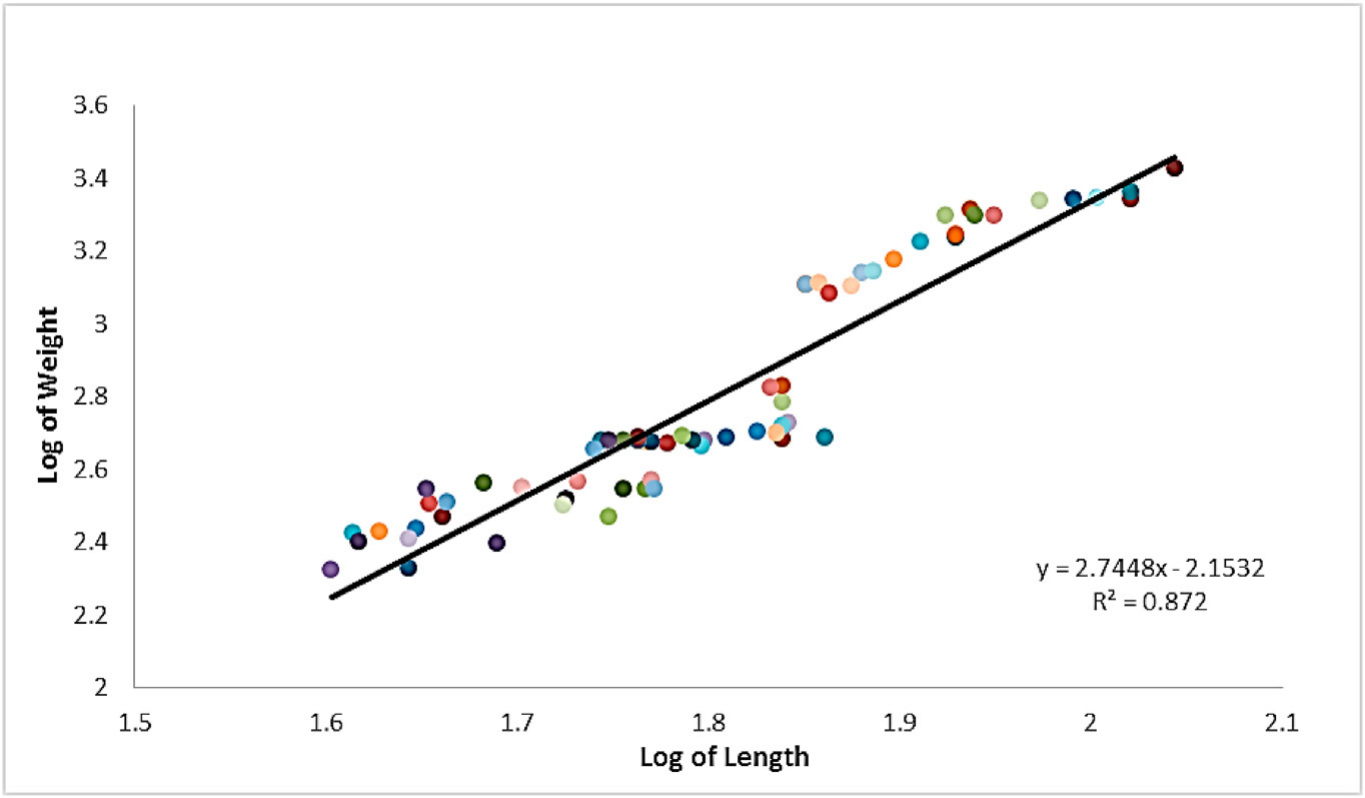
Length-weight relationship of infected *G. niloticus*.

**Figure 3:**
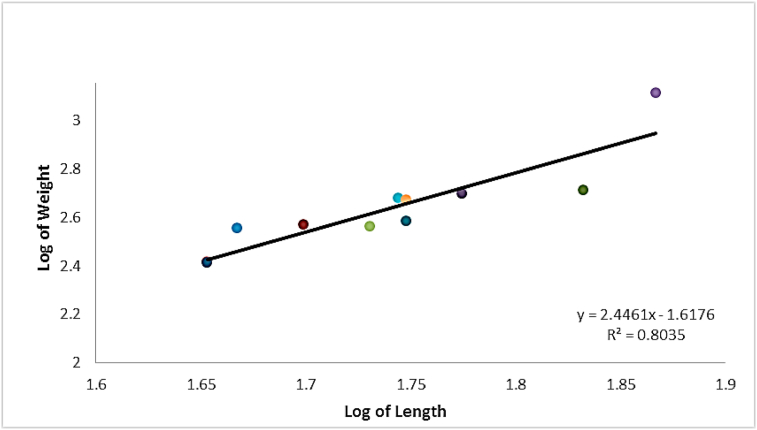
Length-weight relationship of uninfected *G. niloticus*.

The length-weight relationships for the infected fish (Figure 2) and uninfected fish (Figure 3) were logarithmically transformed separately and presented with growth exponent/slope of linear regression curve (b) ranging from 2.75 for infected to 2.42 for uninfected respectively. The fish population is negatively allometric, indicating the slenderness of the fish, characterized by growth exponent <3.

### 3.3 Prevalence parasitic helminth fauna in *Gymnarchus niloticus*

*Raphidascaroides* (nematode) species, named *Nilonema gymnarchi* (philometridae) was recovered from the intestine. Out of 80 total examined fish, 11 (13.75%) were uninfected among which 5 were males and 6 were females (Table 3). The infected fish were 69 (86.25%), comprising of 62 males and 7 females. This totalled to a fish population sample of 67 males (83.75%) and 13 females (16.25%).

**Table 3:** Prevalence of parasitic helminth fauna in relation to the sex of *G. niloticus*.

	Male	Female	Total
Uninfected Individuals	5 (45.45%)	6 (54.54%)	11 (13.75%)
Infected Individuals	62 (89.86%)	7 (10.14%)	69 (86.25%)
Total number Examined	67 (83.75%)	13 (16.25%)	80 (100%)

The intestinal parasite in *G. niloticus* was identified as *Nilonema gymnarchi* (Philometridae) using Akinsanya et al. (2007). The prevalence of the enteric helminth infection concerning the size of *Gymnarchus niloticus* (Table 4) showed that the lengthier individuals were more infected. Fish cohorts within 41–60 cm recorded 1 parasite while fish within the length range of 61–80 cm exhibited an average of 2 parasites, while a further increase to 3 in the parasite prevalence occurred among the fish cohorts of 81–100 cm. There was however an abrupt drop to 0 among the lengthiest fish groups.

**Table 4:** Prevalence of parasitic helminth fauna in relation to the standard length (cm) of *G. niloticus*.

	41–60	61–80	81–100	101–120
Uninfected Individuals	1 (2.38%)	2 (8.00%)	3 (20%)	0
Infected Individuals	41 (97.62%)	23 (92.00%)	7 (80%)	3 (100%)
Total Examined	42 (100.00%)	25 (100.00%)	10 (100.00%)	3 (100%)

### 3.4 Trace metals in environmental media and health

The trend in metal concentrations in surface water recorded was Fe > Zn > Pb > V > Cr > Ni > Cd > Mn > Cu > Co, and that of sediment was Fe > Cr > Mn > V > Zn > Ni > Cu > Pb > Co > Cd (Table 5).

**Table 5:** Metal concentration in surface water and sediment of the Epe lagoon.

Metals	Surface water (mg/L)	FEPA (2003)	Sediment (mg/kg)	FEPA (2003)	Adsorption coefficient
Zinc	0.183 ± 0.04	3.000	**0.672 ± 0.093**	0.012	**3.6**
Cadmium	**0.012 ± 0.01**	0.003	**0.016 ± 0.003**	0.006	**1.3**
Vanadium	0.031 ± 0.003	–	0.863 ± 0.033	–	**27.8**
Iron	**0.586 ± 0.002**	0.300	**528.41 ± 36.6**	0.030	**901.7**
Copper	ND	1.000	**0.226 ± 0.001**	0.025	**–**
Nickel	0.014 ± 0.001	–	0.253 ± 0.006	–	**18.1**
Cobalt	ND	–	0.057 ± 0.001	–	–
Lead	**0.055** ± 0.004	0.010	**0.218 ± 0.027**	0.040	**4.0**
Chromium	0.029 ± 0.010	0.050	39.433 ± 0.12	–	**1,359.8**
Manganese	0.002 ± 0.0003	0.050	**5.703 ± 0.051**	0.030	**2,851.5**

Emboldened concentrations in water and sediment are higher than regulatory limits and emboldened adsorption coefficients are significant. ND = not detected. Sample size (N) = 24.

The concentrations of cadmium (0.01 mg/L) and iron (0.59 mg/L) in the surface water exceeded the limits established by FEPA (2003) which are 0.03 and 0.3 mg/L respectively. The concentration of lead (0.06 mg/L) in the surface water also exceeded the set limit of 0.01 mg/L. The concentrations of zinc (0.67 mg/kg) and cadmium (0.02 mg/kg) were higher than the regulatory limits of 0.012 mg/kg and 0.006 mg/kg set by FEPA (2003). Notably, the concentration of iron markedly exceeded the regulatory limits.

Furthermore, marked sorption from the aqueous phase to the bottom sediment occurred in most of the metals analyzed; in the order of manganese (approximately 2,852 folds)> chromium (approximately 1,360 folds)> iron (approximately 902 folds), vanadium (27.8 folds)> lead (4 folds)> zinc (3.6 folds)> cadmium (1.3 folds).

*Gymnarchus niloticus* has more accumulation potential in the intestine than in the liver and generally accumulated more iron in its tissues than other metals. The trend in metal concentrations in the intestine was Fe > Zn > Cu > Ni > Pb > Cr > Cd > Co > Mn > V (Table 6) and Fe > Zn > Cu > Ni > Pb > Mn > Cr > Co > Cd > V for metal concentrations in the liver (Table 7).

**Table 6:** Bioaccumulation of metals in the parasites and intestine of *G. niloticus*.

Metals (mg/kg)	Concentration			Bioaccumulation				
	Uninfected	Infected		Uninfected		Infected		
	Mean ± SD	Mean ± SD	FEPA	BAF	BSAF	BAF	BSAF	BAF_p/i_
Zinc	5.82 ± 2.66	0.44 ± 0.23	30	32.3	8.7	2.5	0.7	0.7
Cadmium	0.04 ± 0.09	0.003 ± 0.00	0.5	**4**	**2**	0.34	0.15	**12.3**
Vanadium	0.001 ± 0.00	BD	–	0.3	0.001		–	–
Iron	**90.78 ± 47.70**	**7.27 ± 0.13**	0.5	**153.9**	0.2	**12.3**	0.01	0.4
Copper	1.74 ± 1.33	0.42 ± 0.24	3.0	–	**7.6**	–	**1.8**	**2.1**
Nickel	0.29 ± 0.13	0.08 ± 0.03	0.5	**29**	**1.2**	**8.2**	0.3	0.6
Cobalt	0.03 ± 0.07	BD	–	–	0.5	–	–	0.2
Lead	0.19 ± 0.17	BD	2.0	**3.2**	0.9	0.00	–	–
Chromium	0.12 ± 0.15	0.01 ± 0.00	–	**4**	0.00	0.3	0.00	**8.3**
Manganese	0.03 ± 0.05	0.005 ± 0.00	0.50	**15**	0.01	**2.4**	0.00	0.3

Emboldened concentrations of metals in the tissue are higher than regulatory limits and emboldened bioaccumulation factors are significant (>1). BAF_p/i_ = bioaccumulation factor of metals from intestine to parasite. Samples size (N) = 20. BD = below detection.

**Table 7:** Bioaccumulation of metals in the liver of *G. niloticus*.

Metals (mg/kg)	Concentration			Bioaccumulation			
	Uninfected	Infected		Uninfected		Infected	
	Mean ± SD	Mean ± SD	FEPA	BAF	BSAF	BAF	BSAF
Zinc	4.90 ± 0.06	0.14 ± 0.23	30	27.2	7.3	0.8	0.2
Cadmium	0.01 ± 0.0	BD	0.5	**1**	0.63	0.00	0.00
Vanadium	0.002 ± 0.00	BD	–	0.07	0.00	0.00	0.00
Iron	**50.33 ± 7.62**	**3.27 ± 0.13**	0.5	**85.3**	0.1	**5.5**	0.01
Copper	0.75 ± 0.19	0.02 ± 0.01	3.0	–	**3.3**	–	0.1
Nickel	**0.19 ± 0.06**	0.03 ± 0.01	0.5	**19**	0.8	**3**	0.1
Cobalt	0.02 ± 0.01	BD	–	–	0.3	–	–
Lead	0.06 ± 0.17	BD	2.0	**1**	0.9	0.00	–
Chromium	0.04 ± 0.01	BD	–	**1.3**	0.3	0.00	–
Manganese	0.05 ± 0.01	BD	0.50	**25**	0.01	0.00	–

Emboldened concentrations of metals in the tissue are higher than regulatory limits and emboldened bioaccumulation factors are significant (>1). Samples size (N) = 20. BD = below detection.

The concentrations of metals in the intestine of *G. niloticus* were below the regulatory limits, except in the case of iron concentrations which exceeded the established limit in the infected fish while markedly exceeded the limit in the uninfected counterpart (Table 6). Furthermore, notable bioaccumulation factors (BAF) were recorded in the uninfected fish compared to the infected ones. Iron was exceedingly accumulated in the intestine of the infected fish about 154 times higher than the concentration obtained in the surface water. Zinc was also approximately 32 times higher in the intestine of the uninfected fish, relative to the level in the ambient water. Marked BAFs were also recorded for Ni (29 times higher), Mn (15 times higher), Cd and Cr (4 times higher), and Pb (3 times higher). The uninfected fish accumulated Zn > Cd > Cu > Ni significantly from the bottom sediment.

As for the infected fish, on the other hand, significant BAF was only recorded in Fe (12 times higher), Ni (8 times higher), Zn (approximately 3 times higher), and Mn (twice higher). Furthermore, only Cu (1.8) had a significant BSAF among the uninfected fish.

When where the BAF for the fish was significant, the BAF_p/i_ for the parasite was insignificant, vice versa. Where the BAF for the fish was 2.5 (significant), BAF_p/i_ for parasite was 0.7 (insignificant). Conversely, where the BAF for the fish was 0.34 (insignificant), the BAF_p/i_ for the parasite was 12.3 (highly significant). The significant BAFs of 12.3, 8.2, and 2.4 were accompanied by insignificant BAF_p/i_ of 0.4, 0.6, and 0.3 respectively, while the insignificant BAF of 0.3 was accompanied by a significant BAF_p/i_ of 8.3.

In the liver of *G. niloticus,* the concentrations of Fe and Ni exceeded the established regulatory limit among the uninfected fish (Table 7). The uninfected fish markedly accumulated significant concentrations of Fe (approximately 85 times higher), Zn (approximately 27 times higher) and Ni (19 times higher) from the water medium, alongside Cr, Pb, and Cd. They also accumulated zinc and copper from the bottom sediment approximately 7 and 3 times higher respectively than the source.

In the infected fish, on the other hand, only the concentration of iron exceeded the regulatory limit established by FEPA (2003). The group of fish accumulated iron and nickel from the water medium approximately 6 and 3 folds respectively in the liver. The result shows that no metal accumulated in the liver of the infected fish from the bottom sediment.

**Figure 4:**
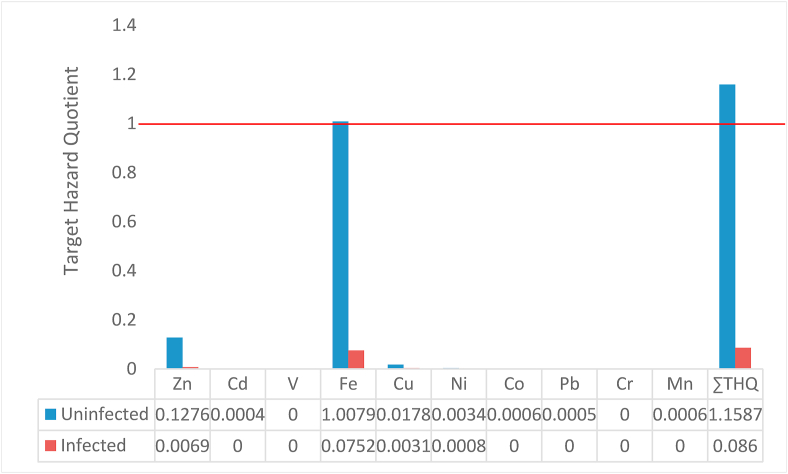
Hazard quotients of metals in uninfected and infected fish. The bars above the red line (≥1) represent significant hazards.

The THQ of iron in the uninfected fish was significant (>1), while that of the infected fish was far below the hazard limit (Figure 4). The combined threat posed by all metals was significant, characterized by the high ∑THQ in the uninfected fish, compared to the low level detected in the infected fish. All other metals in both groups of fish singly posed no health threats whatsoever.

### 3.5 Biochemical biomarkers in *G. niloticus*

The changes in antioxidant response parameters; superoxide dismutase (SOD), catalase (CAT), reduced glutathione (GSH) and lipid peroxidation (Malondialdehyde) in the liver showed a significantly higher stress in the uninfected fish compared to the infected group (Table 8).

**Table 8:** Concentrations (Mean ± SD) of Biochemical markers in the Liver of *G. niloticus*.

Biochemical makers	Uninfected	Infected
Superoxide dismutase (SOD) (μmol/ml/min/mg pro)	2.82 ± 0.401	2.726 ± 0.409
Catalase (CAT) (μmol/ml/min/mg pro)	11.74 ± 0.409	10.194 ± 5.124
Reduced glutathione (GSH) (μmol/ml/mg pro)	0.158 ± 0.036	0.36 ± 0.409
Lipid Peroxidation (MDA) (μmol/ml/mg pro)	0.099 ± 0.029	0.035 ± 0.409

The assessment of the overall relationship between mean biochemical marker activity levels with trace metals concentrations in the *G. niloticus* using the Pearson's correlation coefficient showed that most of the markers had strong correlations with the metals (Table 9). Iron had strong positive correlations with SOD (0.916), CAT (0.948), and GSH (0.961). Zinc, copper, and nickel also exhibited strong positive correlations with SOD (0.919, 0.896, and 0.917), CAT (0.945, 0.945, and 0.946), GSH (0.972, 0.959, and 0.945) respectively (p < 0.05). Strong negative relationships occurred between manganese and SOD, CAT, GSH, and MDA; while chromium had a negative correlation relationship with CAT. A very strong negative relationship also occurred between cadmium and SOD.

**Table 9:** Correlation between biochemical markers and metal concentration in the liver of *G. niloticus*.

	Fe	Zn	Cu	Ni	Pb	Mn	Cr	Co	Cd	V	SOD	CAT	GSH	MDA
Fe	1													
Zn	**0.942**	1												
Cu	**0.980**	**0.979**	1											
Ni	**0.967**	**0.977**	**0.987**	1										
Pb	-0.137	-0.181	-0.216	-0.152	1									
Mn	**-0.542**	**-0.551**	**-0.510**	**-0.573**	-0.11	1								
Cr	-0.312	-0.332	-0.294	-0.266	0.474	0.376	1							
Co	-0.091	-0.198	-0.185	-0.202	0.671	0.256	0.219	1						
Cd	-0.385	-0.328	-0.320	-0.388	-0.123	0.401	-0.07	-0.17	1					
V	-0.231	-0.281	-0.205	-0.313	-0.048	**0.523**	0.367	0.244	0.517	1				
SOD	**0.916**	**0.919**	**0.896**	**0.917**	-0.349	**-0.973**	-0.43	0.129	**-0.96**	0.221	1			
CAT	**0.948**	**0.945**	**0.945**	**0.946**	-0.416	**-0.802**	**-0.69**	0.145	-0.44	0.345	**0.898**	1		
GSH	**0.961**	**0.972**	**0.959**	**0.945**	**-0.546**	**-0.865**	-0.44	0.031	-0.58	0.324	**0.895**	**0.946**	1	
MDA	0.461	0.447	0.439	0.489	-0.414	**-0.608**	0.169	-0.44	-0.34	0.234	0.567	0.249	0.277	1

Emboldened figures represent a significant correlation (p < 0.05).

### 3.6 Histopathological alterations in the intestine of *G. niloticus*

**Figure 5:**
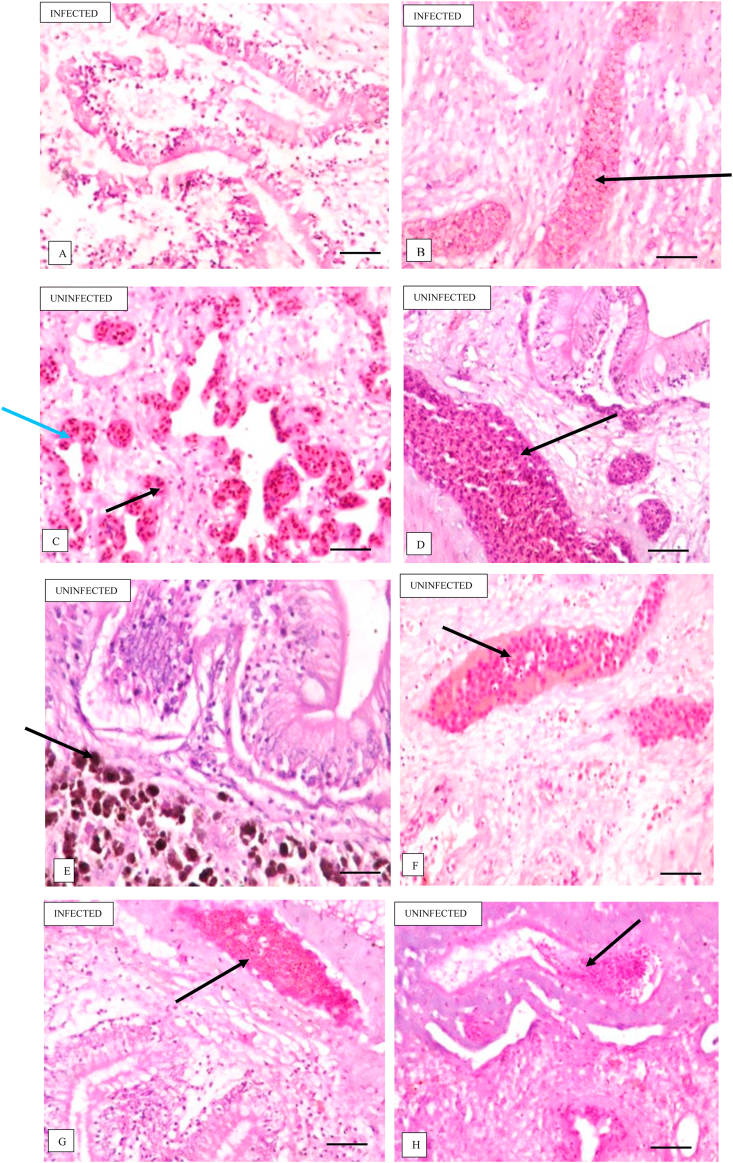
Photomicrographs of histopathological analysis of the intestine of *G. niloticus*. A: Normal villi structure, normal mucosa and submucosa. The normal crypt-villous architecture is well preserved. No significant lesion seen; B: Focal areas of mild vascular congestion (arrow) within the submucosa. However, the villous structure is moderately preserved; C: Severe haemorrhagic lesion (blue arrow) within the villi structure and severe vascular congestion (black arrow) of the submucosa; D: Moderate oedema with an increase in the connective tissue (arrow) of the submucosa, and moderate vascular congestion (arrow) of the submucosa; E: Severe presence of endogenous pigment (arrow) in the submucosa; F: Severe presence of endogenous pigment (arrow) within the submucosa; G: Mild presence of detritus within the lumen and mild vascular congestion; H: Moderate vascular congestion (arrow) within the submucosa. Scale bar = 100 μm.

The histopathological injury observed in the intestinal tissues of the infected fish ranged from the unaltered state (Figure 5 A) to mild vascular congestions in the submucosa (Figure 5 B), and mild presence of detritus in the lumen (Figure 5 G). The uninfected fish on the other hand exhibited more severe injuries, ranging from hemorrhagic lesions and severe vascular congestion (Figure 5 C). Edema, increased connective tissue of the submucosa (Figure 5 D), to vascular congestion (Figure 5D and H) and presence of endogenous pigment in the submucosa (Figure 5E and F).

## 4 Discussions

The contamination of water by metal compounds is a worldwide environmental problem resulting from diverse human activities which tend to continually increase environmental concentrations of these toxicants to levels where the widespread threat to human and animal health results (Pereira et al., 2006). The ubiquity of these metals and their relative importance as pollutants of public concern is brought to the fore by the findings from this study. The physico-chemistry of the water body showed no significant variation in most of the parameters recorded relative to the established limits. The values recorded were within the permissible limits for both FEPA (2003) except for pH with a mean value of 4.48 which was lower than the permissible range of 6.5–8.5 and turbidity which was markedly higher than the established limit. The pH is a very important physicochemical parameter that gives a measure of the acidity or basicity of a water body and in turn influences fish performance in the water. Opadokun et al. (2015) had earlier reported that several fish species stop growing in a low pH which is acidic and lethal in extreme cases. The low pH values recorded in the lagoon with levels ranging from 4.30 – 4.66 are indicative of high acidity and may enhance the bioavailability of toxicants, which may be further aggravated by the high turbidity.

The importance of length – weight relationship and condition factor (k) in fisheries biology cannot be overemphasized as values obtained from such assessments are useful in assessing the relative well - being of a fish population as compared to others of the same species exposed to the same or different conditions such as food availability, density pathogens or toxicants.

The prevalence of the male fish over the female in the population is deviant from the expected normal sex ratio of male to female 1:2. The main factor likely responsible for the anomaly could be the fishing techniques adopted by the fishermen. The condition factors of the infected (0.252) and uninfected (0.268) fish groups didn't vary significantly. Furthermore, the growth exponent suggests negative allometry in both groups; marked by the graph slope <3 which indicates slenderness and unfitness possibly due to environmental stressors such as toxicants (Ikongbeh et al., 2012). The growth exponent of the infected fish: 2.74 was however higher than that of the uninfected: 2.45, suggesting that the former might have adapted to some factors which made them cope better in the perturbed environment. Falaye et al. (2015) earlier observed negative allometric growth among a sampled population of *G. niloticus* in the same lagoon across dry and wet seasons. They also recorded K-values of 0.711 ± 0.3485 and 0.7047 ± 0.507, which were all <1. The condition factors of the fish in the current study also compare to those of Odo et al. (2013) who observed mean value of 0.78 ± 0.11 for *G. niloticus* in the floodplain of River Anambra, Southeast Nigeria.

The study conforms to the work of Akinsanya et al. (2007) who earlier recovered two nematodes: *Raphidascaroides* species (Heterocheillidae) and *Nilonema gymnarchi* (Philometridae) from the intestine of *G. niloticus*. The helminth parasites *Nilonema gymarchi* and *Thwaitia bagri* are the two representative genera of the family Philometridae in Africa.

Prevalence of the parasitic helminth relative to sex showed a higher susceptibility in the infected male fish (89.86%) than the female counterpart (10.14%). This result is at variance with that of Akinsanya et al. (2007) who detected no infection in males while the female specimens had a prevalence of 50% and so concluded that egg formation and gestation might have played some roles in suppression of the immunity of the females. In the current study, however, the higher susceptibility in the males may be due to encounter with parasites as they explore various parts of the lagoon for food and mates. There have been a varied explanation of the relationship between parasite susceptibility and sex by various Authors (Olurin et al., 2012). According to Emere (2000) the difference in the prevalence of parasitic helminth infection in male and female fish can be attributed to the feeding habit, particularly the quality and quantity of food. He further stated, that due to the physiological state of female fish, most gravid females may have reduced resistance to parasitic infections.

Although only Fe exhibited a significant hazard quotient, in the light of FEPA (2003) established limits, impermissible concentrations of Cd and Pb in the water of the lagoon may also pose sub-lethal health and/ecological concerns. The concentrations of Zn, Cd, Fe, Cu, Pb, and Mn in the sediment also exceeded the safe limits recommended by FEPA (2003) and this is attributable to the attendant sorption capacities recorded for the metals, particularly in Fe, Cr, and Mn where extreme sorption capacities were detected. The anthropogenic activities may release metals in the water body through surface runoff from point and non-point sources in the catchment area. The metals in the overlying water column are liable to precipitate to the bottom over time. The high concentrations of metals in the sediment may therefore be due to the repository nature of the bottom sediment (Akinsanya, 2020). Saliu and Akinsanya (2014); Ukwa et al. (2015); and Akinsanya et al. (2020) have reported that metal concentrations in the bottom sediment of the lagoon exceeded WHO-approved limits. The persistent marked sorption of the trace metals to the bottom sediment, particularly Fe, Cr, and Mn may mean danger for a benthopelagic fish*.* Worse still, future re-pollution of the overlying water phase by the bottom sediment may occur after recuperation. The non-biodegradability and accumulative potential of metals make them persistent and increases their environmental health impacts.

Comparison of the BAF of metals in uninfected fish with the infected counterparts showed that the former accumulated more metals and at higher rates than the latter. Results showed that Zn (32 folds), Cd (4 folds), Fe (154 folds), Ni (29 folds), Pb (3 folds), Cr (4 folds), and Mn (15 folds) all bioaccumulated in the intestine of the uninfected fish, compared to the infected fish which merely bioaccumulated Zn (about 3 folds), Fe (12 folds), Ni (8 folds), and Mn (4 folds) only. Significant variability also occurred in the indices of BSAF between the two groups. The uninfected fish accumulated Zn (8.7), Cd (2), Cu (7.6), and Ni (1.2) from the bottom sediment, while the uninfected only accumulated Cu (1.8). Similarly, although at a lesser rate, the BAF and BSAF of the metals the liver of the uninfected fish were higher than the infected fish. Zn (27.2), Cd (1), Fe (83.3), Ni (19), Pb (1), Cr (1.3), and Mn (25) had much higher BAF indices in the uninfected fish than the infected counterparts, which were Fe (5.5) and Ni (3) only. Furthermore, the uninfected fish accumulated Zn (7.3) and Cu (3.3) from the bottom sediment, while no significant BSAF was recorded in the infected counterparts. A notable observation is that although the concentrations of the metals were higher in the sediment than in the overlying water medium, the fish however bioaccumulated more metals from the water phase than from the bottom sediment. Metals are liable to accumulate in the fish as it releases an electric field which is conveyed in the water for detection of prey, predators, and mates.

The current observations conform to the work of Ukwa et al. (2015) who studied the trace metal accumulation in three catfish species of the lagoon *(Malapterurus electricus, Chrysichthys nigrodigitatus* and *Synodontis clarias*) as well as Akhiromen and Ogbonne (2018) who recorded high concentrations of Fe, Cu, and Zn in the intestinal tissues of *Macrobrachium vollenhovenii* of the Lagoon.

The concentration of trace metals in fish is a function of an interplay of factors such as the foraging behaviour of the organism (Obasohan and Oronsaye, 2004), trophic status, source and concentration of metal, and presence of other ions in the milieu (Isibor et al.*,* 2020),biomagnification of a the metal (Barlas, 1999), presence of metallothioneins, and other intracellular metal ligands (Deb and Fukushima, 1999), temperature (Isibor, 2017), species, age, sizeand the metabolic rate of the animal (Isibor et al.*,* 2016).

Marked bioaccumulation of Zn, Fe, and Mn may be due to their thresholds of essentiality which necessitate their moderation for vital physiological functions and homeostasis (Chen and Chen, 1999). On the contrary, the non-essential elements have no biological function rather toxic effects even at low concentrations, hence they are spontaneously excreted through the gills, bile, kidney, and skin.

In this study, only the concentration of Fe exceeded the established limit of FEPA (2003), while the limits for concentrations of Fe and Ni were exceeded in the liver. The induction of metallothionein occurs in the liver tissue of fish. Metallothioneins have high affinities for metals, hence they readily bind up to their molecules (Isibor et al.*,* 2016). The presence of parasites in the intestine may have also sequestered the toxicant burden in the organ, compared to the liver which harbored no parasites. Similar findings of higher metal accumulation in the intestine than the liver have been reported by Shanti et al. (2000), Ahmed et al. (2009), Ishaq, et al. (2011), and *Zhang* et al. (2018). The Authors submitted that the main accumulation tissues for wild fish are the bladder and intestine which supports the current observations, thus suggesting that the intestine of the fish may represent good bio-monitor of metals in the lagoon (Falusi and Olanipekun, 2007; Isibor and Imoobe, 2017).

Linking up the higher accumulation of metals in uninfected fish than the infected with the results of higher up-regulation of biochemical indicators in the former than the latter suggests that metals might have induced reactive oxygen species (ROS) in the absence of parasites (Akinsanya et al.*,* 2020). Therefore, continuous accumulation of these metals may result in heightened ROS in the uninfected fish beyond the threshold of tolerance, thereby culminating in oxidative stress and physiological imbalance, particularly in the uninfected fish (Akinsanya et al.*,* 2019). Correlation analysis further buttresses the strong relationship between metal concentrations and the activities of the stress biomarkers in both fish groups (Isibor et al.*,* 2020).

Metal induced ROS are detoxified by a set of antioxidant enzymes that protect macromolecules such as proteins, lipids, and nucleic acids against damage (Lushchak et al. 2001; Ozmen et al. 2004). These antioxidant enzymes have been shown to work in a cooperative or synergistic manner to protect against oxidative stress and tissue-specific damage. Thus, the enzyme systems are suitable biomarkers for reactive oxygen species (ROS) and as a potential tool in environmental risk assessment, since they defend against exposure to stressors (Kohen and Nyska, 2002). The earliest studies on toxicant-induced oxidative stress bioindication are observed as shifts in activities of the biochemical defense systems; including enzymatic activities such as SOD, CAT, GPx and GST and non-enzymatic activity such as GSH (Vijayavel et al., 2004).

SOD is the first enzyme to respond against oxygen radicals and offers the stongest response against oxidative stress (Wright and Pamela, 2002) by accelerating the dismutation of superoxide (O_2_^-^) to H_2_O_2_ which damages the membrane and biological structures (Vijayavel et al., 2004). The SOD level was higher in the uninfected fish than the infected ones supports the indications deduced from the bioaccumulation analysis. Moreover strong positive correlations between SOD and most of the metals- Fe (0.916), Zn (0.919), Cu (0.896), and Ni (0.917) suggests that the metals may have inflicted more toxicity in the uninfected.

Catalase (CAT) being one of the most efficient antioxidant enzymes which does not get overwhelmed by stressor. CAT works in tandem with SOD and it reacts with H_2_O_2_ to form water and molecular oxygen. CAT level in the uninfected fish was also higher than the infected, further suggesting that the infected fish possibly coped better with the stressor. This observation is in line with the findings of Saliu and Bawa-Allah (2012) who stated that an inhibition of the enzyme SOD will expectedly result in a reduction in the activity of the enzyme CAT, due to a decrease in H_2_O_2_ generation from SOD activities. Strong positive correlations also occurred between CAT and Fe (0.948), Zn (0.945), Cu (0.945), and Ni (0.946). GSH also exhibited strong positive relationships: 0.961, 0.972, 0.959, and 0.945 respectively with the metals.

Lipid peroxidation as expressed by the MDA (Akinsanya et al.*,* 2020) showed a weak positive correlation with metals in the liver tissues except for Pb, Mn, Cr, Co, and Cd which were negatively correlated with the lipid peroxidation product. Among all, only Mn (-0.608) has a significant negative relationship with MDA. Lipid peroxidation expresses the oxidative damage in a biological system (Akinsanya et al.*,* 2019). Oxidative damage becomes evident when there is no equilibrium between the reactive oxygen species (ROS) generated as a result of bioaccumulation of the trace metals and the antioxidant biomarker response. Alternatively, the ROS overwhelm the production of antioxidant biomarkers. The elevated lipid peroxidation concentration observed in the uninfected fish compared to the infected may be as a result of the absence of parasites in the former to share the burden of the trace metals. The microsomal metabolism of xenobiotics and microsome mediated redox cycling which gives rise to oxyradicals are capable of oxidizing membrane lipids (Akhiromen and Ogbonne, 2018).

The histopathological comparisons made between the uninfected and infected fish showed consistency with the outcomes of other comparisons made in this study. These evidence were marked by tissue alterations in the infected fish ranging from no observed changes to mild alterations, while the uninfected exhibited more severe tissue injuries such as hemorrhagic lesions, severe vascular congestion, edema, the increased connective tissue of the submucosa, and vascular congestion.

## 5 Conclusion

Although the physicochemical parameters of the lagoon appeared to be in fair conditions, the *G. niloticus* in the lagoon however exhibited notable sub-lethal toxicity effects. This implies that mere assessment of the physico-chemistry may not provide the true picture of the ecological condition of aquatic habitat. The better-coping potentials exhibited by the parasitized fish than the non-parasitized, coupled with the significant bioaccumulation interferences exhibited by the parasite *Nilonema gymnarchi* is an indication that the parasite may be a good metal sequestration agent for the fish and can be used to forestall the significant health hazard quotient posed by the current level of iron and the synergy of all metals analyzed in the lagoon.

In light of the ongoing industrial developments springing up in the catchment vicinity of the lagoon. It is recommended that regulators ensure strict compliance with and enforcement of environmental best practices in the treatment and disposal of wastes generated by industries within the area. Continuous monitoring of the lagoon is strongly recommended.

## Declarations

### Author contribution statement

P.O. Isibor, B. Akinsanya, and S.J. Kayode: Conceived and designed the experiments.

P.O. Isibor, B. Akinsanya, T. Sogbamu, F. Olaleru, A. Excellence, B. Komolafe and S.J. Kayode: Performed the experiments.

P.O. Isibor, T. Sogbamu, F. Olaleru, and A. Excellence: Analyzed and interpreted the data.

P.O. Isibor, F. Olaleru, and A. Excellence: Contributed reagents, materials, analysis tools or data.

P.O. Isibor: Wrote the paper.

### Funding statement

This research did not receive any specific grant from funding agencies in the public, commercial, or not-for-profit sectors.

### Competing interest statement

The authors declare no conflict of interest.

### Additional information

No additional information is available for this paper.
